# Successful treatment of metastatic dermatofibrosarcoma protuberans of the breast to the lungs with imatinib mesylate: a case report

**DOI:** 10.3332/ecancer.2025.1912

**Published:** 2025-05-27

**Authors:** Stephen Kpatsi, Joseph Daniels, Kofi Adesi Kyei, Verna Vanderpuye

**Affiliations:** 1 National Radiotherapy, Oncology and Nuclear Medicine Centre, Korle Bu Teaching Hospital, Accra, Ghana; 2 Department of Radiography, University of Ghana, Legon, Accra, Ghana; a https://orcid.org/0009-0005-8954-0509; b https://orcid.org/0000-0002-1466-150X; c https://orcid.org/0000-0003-3485-5368; d https://orcid.org/0000-0003-3656-6965

**Keywords:** imatinib mesylate, lung metastasis, dermatofibrosarcoma protuberans, complete response, DFSP, dermatofibrosarcoma protuberans

## Abstract

Dermatofibrosarcoma protuberans (DFSPs) is a low-grade mesenchymal tumour of dermal origin. Although lung metastasis is common, primary breast involvement is a very rare occurrence. The head and neck region, trunk and extremities are the common primary sites even though any site of the body may be potentially involved. Complete surgical resection with negative margins is the standard of care for nonmetastatic cases of DFSP. To the best of our knowledge, there have been no previous reports of metastatic DFSP of the breast to the lungs among male patients in sub-Saharan Africa. This case report presents data on a 32-year-old male with metastatic DFSP of the left breast to the lungs who was successfully treated with 800 mg daily imatinib mesylate therapy and achieved a complete radiological and clinical response after 1 year of palliative therapy.

## Introduction

Dermatofibrosarcoma protuberans (DFSPs) is a low-grade mesenchymal tumour of dermal origin [1]. DFSP is a very rare soft tissue sarcoma that accounts for <1% of all soft tissue sarcomas and <0.1% of all malignant neoplasms. The annual global incidence is estimated to be about 4.2–4.5 per million cases [2]. The most common site of occurrence is the trunk (57%), followed by the proximal extremities (23%) [3]. Breast involvement is not common although previously documented [4]. Complete surgical resection with negative margins is the standard of care for nonmetastatic cases of DFSP.

Adjuvant radiation therapy is indicated for patients with positive, close or indeterminate margins after primary surgery whereas patients with high-grade histology or fibrosarcomatous differentiation require adjuvant systemic therapy. These factors as well as high mitotic rates and increased cellularity are associated with decreased survival [5]. Metastatic or inoperable patients are treated with palliative systemic and/or radiation therapy depending on various factors such as performance status, symptoms and comorbidities of the patient.

To the best of our knowledge, there have been no previous reports of DFSP of the breast with lung metastasis among patients in sub-Saharan Africa. This case report presents data on a single patient with metastatic DFSP of the breast to the lungs who was successfully treated with palliative imatinib therapy and achieved a complete radiological and clinical response after 1 year.

## Case presentation

We present the case of a 32-year-old male who was first seen in January 2021 for treatment at a major cancer treatment centre in Accra, Ghana. He was in his usual state of health until early 2019 (2 years prior to presentation) when he noticed a small growth in the left breast which gradually increased in size over time. He reported to a peripheral health facility where the mass was surgically excised. Histopathological examination showed sections of the tumour composed of mildly pleomorphic spindle cells in fascicles with a storiform pattern infiltrating surrounding subcutaneous tissue in a honeycomb manner. Mitoses were focally increased (5/10 HPF). These features were consistent with DFSP. Tumour infiltration of the dermis of the overlying skin was also noted with the involvement of the margins of the pieces of tissue submitted.

Because of positive microscopic margins, the patient was recommended to have a local re-excision; however, he was lost to follow-up. Five months later (middle of 2019), he developed a local tumour recurrence in the left breast wall and resorted to traditional herbal remedies which proved ineffective with increasing tumour size and copious offensive discharge.

**Figure 1.**
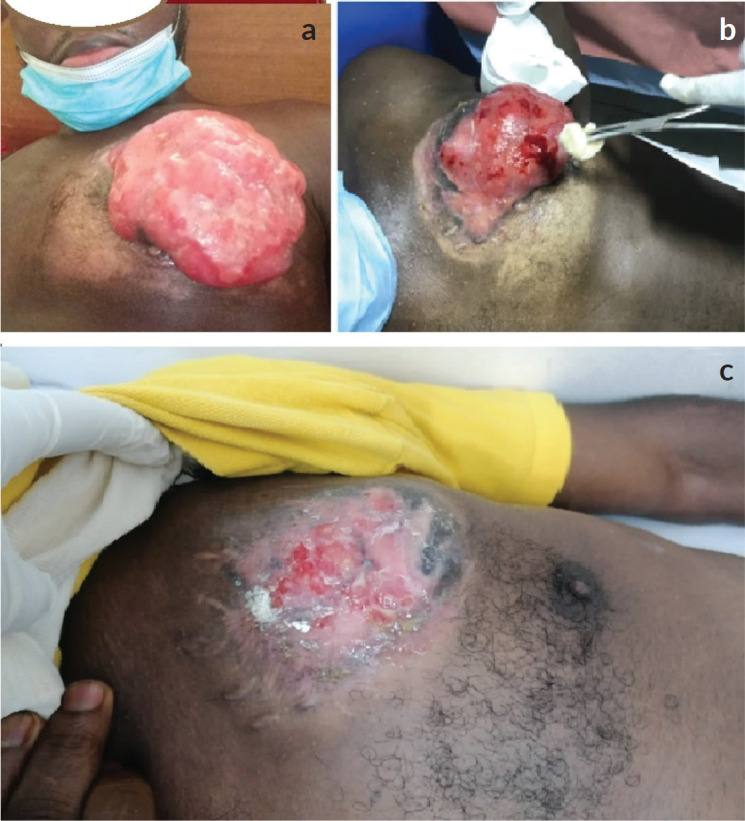
Local tumour of the patient at presentation, 2 months and 6 months after starting treatment. (a): The gross tumour of the left breast prior to the commencement of antineoplastic therapy, 13.6 × 12.0 × 8.3 cm in size. The tumour was multinodular with a foul smell and offensive discharge but no active or contact bleeding. (b): Residual tumour after 2 months of treatment with tab imatinib, 800 mg daily. There was a drastic reduction in the size of the primary tumour. (c): Significant regression after 6 months of systemic therapy.

In January 2021, he presented at our centre with complaints of a left breast mass with foul-smelling discharge and chest pain/discomfort. The patient had no significant past medical history or comorbidities. He was a father of two children with no history of tobacco smoking or alcohol intake. On physical examination, he was generally healthy looking with a performance status of ECOG 1. There was a huge ulcerated multi-lobulated nodular left breast mass with no active bleeding measuring 13 × 12 × 7.5 cm. The tumour was attached to the chest wall. There was no palpable axillary, cervical or supraclavicular lymphadenopathy. Serum electrolytes and blood urea and nitrogen (BUN), creatinine were within normal range. Complete blood counts showed an absolute white blood cell count of 10.65 × 10^9^/L, an absolute neutrophil count of 7.36 × 10^9^/L and a hemoglobin level of 9.5g/dL. A chest computed tomography (CT) scan showed a left breast mass measuring 13.6 × 12.0 × 8.3 cm with features of malignancy and local spread to the left axillary lymph nodes, the largest of which measured 2.0 × 1.5 cm. The tumour infiltrated the underlying pectoralis major muscle. There were few bilateral lung infiltrates and atelectasis with mediastinal metastatic lymphadenopathy. The extent of the atelectasis was moderate, primarily affecting the lower lobes of both lungs. The infiltrates appeared more pronounced in the left lower lobe, where a significant area of consolidation was noted, contributing to the partial collapse of the lung tissue. The patient was diagnosed with metastatic DFSP of the left breast to the lungs with ipsilateral axillary and mediastinal lymph nodes. The management of this patient was discussed extensively by a multidisciplinary tumour board, which included specialists in oncology, surgery, radiology and pathology. This collaborative approach ensured a comprehensive evaluation of the patient’s condition and contributed to the decision to initiate targeted therapy. The patient was started on 800 mg of Imatinib mesylate daily. Figure 1 illustrates the local tumour of the patient prior to starting treatment, at 2 and 6 months after starting treatment. Three weeks following the start of treatment, the patient reported a significant reduction in the pain and discomfort he experienced in his chest. The intensity of the unpleasant smell emanating from the ulcerated tumour had reduced considerably. After 2 months of treatment, there was a 50% reduction in the size of the left breast mass. His performance status had improved to ECOG 0.

**Figure 2.**
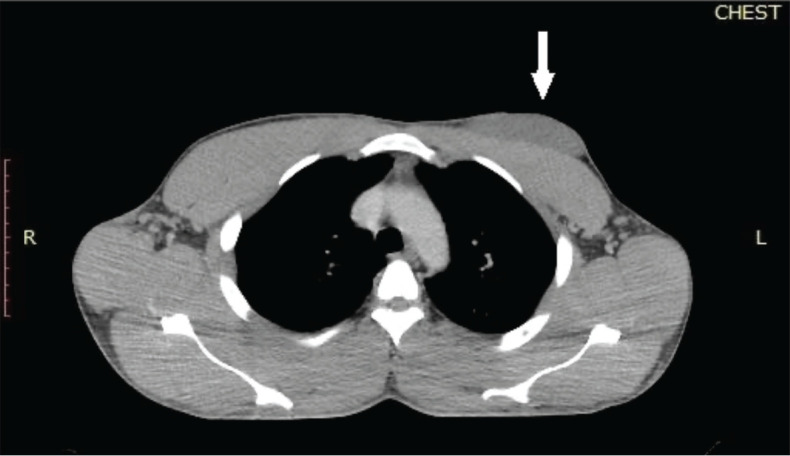
Chest CT scan of the patient which was done after 3 months of systemic treatment. The chest window of a CT scan of the patient showed a hypodense mass located anteriorly to the pectoralis major muscle. The white arrow indicates the residual left breast lesion which was abutting but not infiltrating the underlying chest wall muscle. The size of the tumour was 8.4 × 8.0 × 5.4 cm. The tumour extended from the left parasternal line to the left anterior axillary line.

**Figure 3.**
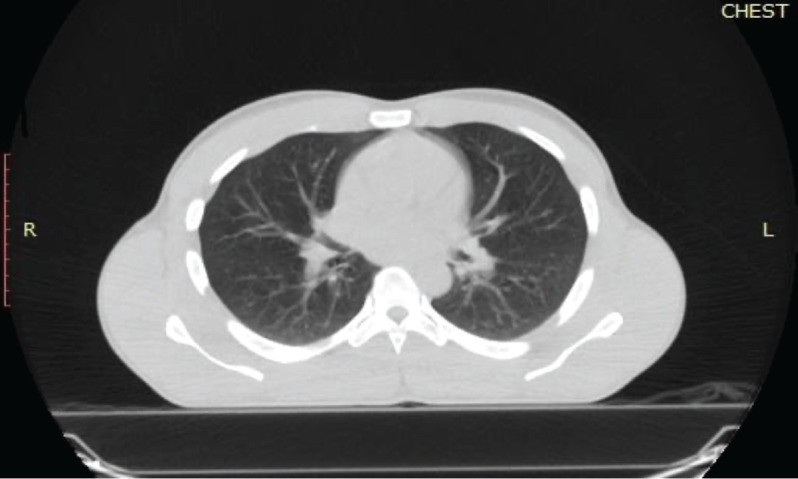
Reassessment chest CT scan of the patient. The lung window of a chest CT scan of the patient was done after 3 months of systemic therapy. It shows bilateral lung fields with no evidence of atelectasis or pulmonary nodules.

A repeat chest CT scan done after 3 months of treatment in May 2021 showed a 74% partial response to treatment. There was a residual left breast mass of 8.4 × 8.0 × 5.2 cm down from the pretreatment size of 13.6 × 12.0 × 8.3 cm as seen in Figure 2. All the pulmonary lesions as well as the mediastinal and axillary lymphadenopathies had resolved Figure 3. The patient continued Imatinib on account of the complete radiological response of the metastatic lesions and partial response of the primary tumour.

**Figure 4.**
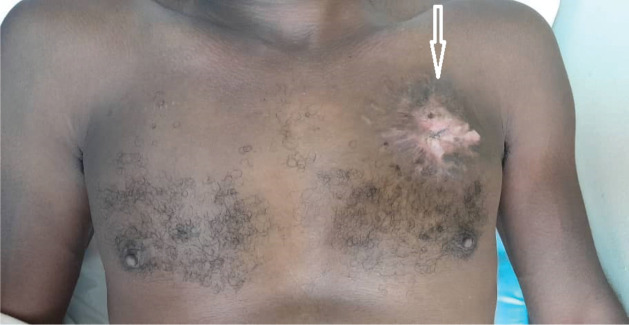
The chest wall of the patient after 24 months of therapy. The white arrow shows the tumour site on the left side of the chest wall of the patient. There was extensive scar tissue without protrusion of the left chest wall 24 months after starting palliative systemic therapy with imatinib mesylate.

Clinical reassessment in June 2022, showed a 6 × 5 cm firm area of indurated skin above the left nipple line shown in Figure 4. After 1 year of Imatinib therapy, clinical examination revealed a complete clinical response of the primary tumour with only scar tissue at the local site. There was no pain or tenderness. A CT scan of the chest also revealed no abnormal pulmonary or mediastinal findings. The patient is still on Imatinib therapy and was last seen for review in June 2023, still in remission with no evidence of the disease recurrence. The patient reported no side effects of Imatinib.

### Patient’s perspective

After the initial surgery with positive resection margins, the patient defaulted on re-excision partly due to fear of having another surgery. Following the recurrence of the tumour, he resorted to alternate treatment due to financial constraints. At the peak of the disease, the patient was unable to wear shirts or other upper garments of his size. Initially, the severity of the pain and the foul smell of the tumour were his most important concerns. After 6 months of treatment, both had completely resolved. He was able to return to work after 10 months.

## Discussion

DFSP exhibits a unique morphology characterised by a homogenous population of neoplastic spindle cells arranged in a storiform pattern amidst a fibrous stroma. Even though specific immunohistochemical markers for DFSP are lacking, the tumour cells typically demonstrate positivity for CD34 and vimentin on immunohistochemistry analysis [6]. Pathological examinations, including biopsy and immunohistochemical analysis, are essential in definitively establishing the diagnosis of DFSP. These examinations help in minimising misdiagnosis by differentiating DFSP from other benign or malignant skin conditions such as cutaneous leiomyosarcoma, cellular fibrous histiocytoma (dermatofibroma), peripheral nerve sheath tumours and, myxoid or synovial sarcoma.

The scenario of incomplete histopathological workup is typical for many patients in resource-limited settings where the cost of histopathological analysis must be paid for out of pocket. As a result, patients often have to choose between a full diagnostic workup and a limited workup with only tests and investigations that are of direct therapeutic consequence.

Imatinib mesylate is a tyrosine kinase inhibitor that has been approved by the US Food and Drugs Authority for the management of recurrent, unresectable or metastatic DFSP in adults [7]. This patient received the required dosage of Imatinib mesylate at no cost under the kind auspices of the Max Foundation through the Glivec International Patient Assistance Program. The drug would have otherwise not been available to or affordable for this patient. The primary prospective data regarding imatinib’s efficacy in treating advanced DFSP stems from a combined analysis of two Phase II trials undertaken by the European Organisation for Research and Treatment of Cancer and the Southwest Oncology Group. These trials involved a collective cohort of 24 patients diagnosed with locally advanced or metastatic DFSP. The patients received varying doses of imatinib, ranging from 400 to 800 mg [8]. Even though a daily dosage of 800 mg is currently recommended in the metastatic setting, a recent analysis in India involving 14 patients did not find any significant difference in clinical outcomes with a daily dose of either 400 or 800 mg [9].

This case is interesting because it is one of the few reported successful cases of patients with metastatic DFSP treated in low-resource settings. DFSP is known to have a high local recurrence rate but a low rate of distant metastasis (1%–4%) [10]. Paradoxically, patients in resource-limited settings are often diagnosed at the metastatic stage when the treatment intent is palliative. The median survival of patients with metastatic DFSP is about 2 years. Higher mortality rates have been associated with high mitotic rates, increased cellularity, male sex and location of the primary tumour on the head, neck or extremities [11].

There are many reasons for late presentation, diagnosis and treatment among cancer patients in low-middle-income countries. Common, reasons are the cost of therapy, fear of the recommended treatment procedure and a false sense of good health after the surgical removal of the gross tumour. The occurrence of treatment-induced toxicity can also pose compliance challenges for some patients. Fortunately, our patient did not experience any severe side effects. He was particularly motivated to continue therapy following a drastic reduction in the size of the primary tumour as early as 2 months after the commencement of the treatment.

Our patient had a large primary tumour size of 13.6 cm in the widest dimension. A recent population-based study demonstrated that the age at diagnosis (> 50 years), histologic grade III and tumour size ≥ 10 cm were negative independent risk factors for cancer-specific mortality whereas tumour localisation and surgical procedures had no significant impact on survival [11].

## Conclusion

A complete histopathological diagnostic workup is not always performed for patients in low-resource settings with DFSP. After the diagnosis of cancer, patients face a complex set of challenges that may ultimately result in a delay in commencing treatment. Metastatic DFSP can be successfully treated with imatinib mesylate.

## Conflicts of interest

The authors declare that they have no conflicts of interest.

## Funding

The authors declare that no funds, grants, awards or other support were received either during the conduct of the study or during the preparation of the manuscript.
